# An Evidence-Based and Mechanistic Approach to Reducing the Risk of Anterior Cruciate Ligament Injury: An Exercise and Sport Science Australia Position Statement

**DOI:** 10.1007/s40279-026-02450-3

**Published:** 2026-05-14

**Authors:** Tyler J. Collings, Laura E. Diamond, Rod S. Barrett, David J. Saxby, David G. Lloyd, Rod Whiteley, Brooke E. Patterson, Kay M. Crossley, Matthew N. Bourne

**Affiliations:** 1 Australian Centre for Precision Health and Technology (PRECISE), Griffith University, 1 Parklands Dr, Southport, Gold Coast, QLD 4215 Australia; 2 School of Allied Health, Sport and Social Work, Griffith University, Gold Coast, QLD Australia; 3 Rehabilitation Department, Aspetar Orthopaedic and Sports Medicine Hospital, Doha, Qatar; 4 School of Allied Health, Curtin University, Perth, WA Australia; 5 La Trobe University Sport and Exercise Medicine Research Centre, La Trobe University, Melbourne, VIC Australia; 6 Australian IOC Research Centre, La Trobe University, Melbourne, VIC Australia

## Abstract

Anterior cruciate ligament (ACL) rupture is a serious sporting injury associated with impaired performance, high recurrence rates, early-onset knee osteoarthritis and substantial health and economic burden. In Australia, ACL injury incidence has increased over the past two decades, with particularly high rates observed among female and adolescent athletes participating in cutting and landing sports. Neuromuscular and structured warm-up programs can reduce ACL injury risk; however, implementation and adherence remain inconsistent, and evidence for effectiveness in males, elite athletes and several high-risk sports is unclear. Practitioners, therefore, require clear, evidence-based guidance to prescribe effective prevention strategies across diverse populations. This position statement provides contemporary, evidence-informed recommendations to guide exercise prescription for ACL injury prevention by (1) critically evaluating the effect of existing exercise interventions on ACL injury incidence, and (2) synthesising evidence on intrinsic risk factors and mechanistic pathways for non-contact ACL injury to inform targeted training strategies. Two systematic literature searches were conducted in Scopus, Embase and PubMed. The first identified randomised and non-randomised controlled trials and cohort studies evaluating the effect of exercise-based interventions on ACL injury incidence. The second identified prospective studies examining intrinsic risk factors for non-contact ACL injury. Evidence on ACL injury aetiology was synthesised by integrating injury risk factors, video analyses of injury events, in vivo and in vitro laboratory studies and in silico biomechanical modelling. We propose a tiered framework for exercise prescription. Tier 1 strategies emphasise population-level neuromuscular and structured warm-up programs, which meta-analyses indicate can reduce ACL injury risk by approximately 60% in adolescent female soccer, basketball and handball players. Effective programs typically combine plyometric, strength, balance/proprioception, mobility and agility exercises performed for 10–30 min, 2–3 times per week. Tier 2 strategies target specific neuromuscular deficits or high-risk movement patterns, including low knee flexion, dynamic knee valgus, lateral trunk flexion and excessive step width. Interventions may include plyometric and resistance exercises, motor control drills and neurocognitive challenges to increase transfer to game scenarios; however, direct evidence linking Tier 2 strategies to reduced injury incidence remains limited. This position statement provides an evidence-based framework to support the design and practical implementation of effective ACL injury prevention strategies in sport.

**Supplementary Information:**

The online version contains supplementary material available at https://doi.org/10.1007/s40279-026-02450-3.

## Key Points

**Table:** 

Neuromuscular training and warm-up programs are effective in reducing ACL injury risk, particularly in young females; however, adherence to these programs is often low, and evidence supporting their effectiveness in males, elite athletes and sports other than soccer is limited.
Population-wide neuromuscular training programs should form the foundation of ACL injury prevention efforts. Individualised training approaches may be considered when specifically designed to target established injury mechanistic pathways and causal risk factors.
Targeted training strategies should prioritise improvements in body posture and neuromuscular control to reduce external knee loads and increase muscular support to assist the ACL in stabilising the knee.

## Background

Anterior cruciate ligament (ACL) rupture is a devastating sporting injury and Australia has the highest rate of ACL reconstruction surgery in the world (77.4 per 100,000) [1]. After returning to sports following ACL reconstruction, many athletes are unable to achieve their pre-injury level of performance [2] and often display pronounced deficits in muscle strength [3], power [4] and size [5], in addition to reduced psychological well-being [6]. Up to one-third of athletes then go on to sustain a second ACL rupture, either to the same or opposite knee, resulting in further time loss and increased likelihood of ceasing sports participation altogether [7]. Long-term, ACL injury also results in an elevated risk of early-onset knee osteoarthritis [8, 9]. Collectively, these adverse outcomes impose a significant health and economic burden [10].

**Fig. 1:**
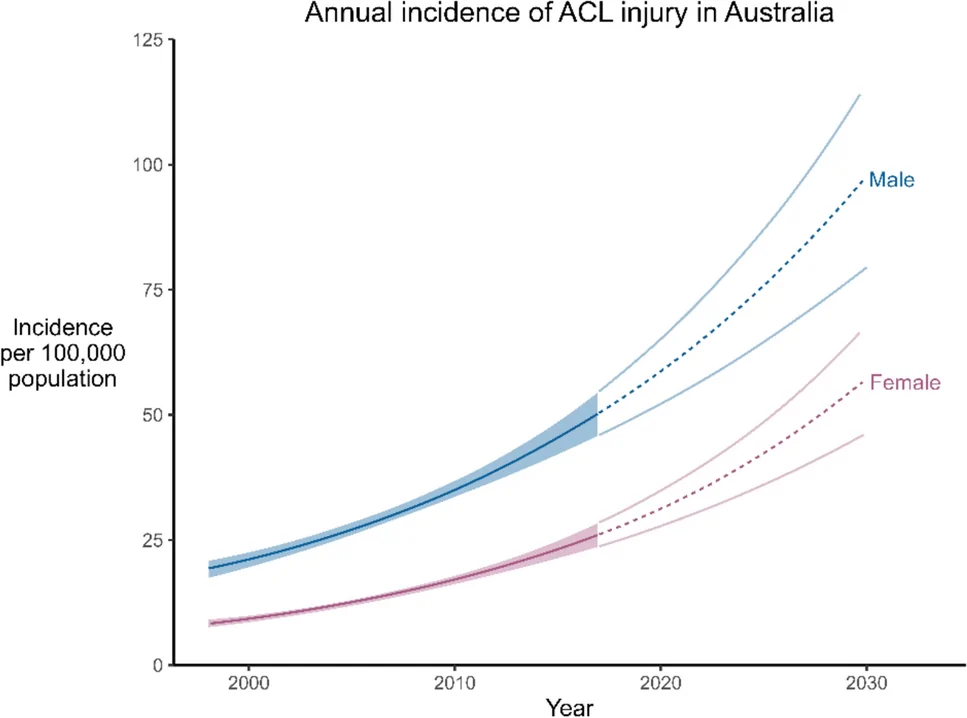
Annual incidence (per 100,000 individuals) of anterior cruciate ligament (ACL) injury in Australia for males and females diagnosed by hospitals between 1998 and 2018. Data are modelled using a negative binomial regression model and 95% confidence interval. The solid shaded regions indicate reported data, and the transparent regions indicate forward predictions for 2018 to 2030. Adapted from Maniar et al. [11] under a Creative Commons license

Despite a proliferation of research, male and female ACL injury rates have increased annually in Australia since 1998 (Fig. 1), with the largest increases observed among females and adolescent athletes aged 5–14 years [1, 11]. Female athletes are 3–8 times more likely to sustain an ACL injury than males, depending on the sport [12]. Several factors may contribute to these rising population-level ACL injury rates, including increased participation in high-risk sports, greater demands of training and competition, reduced physical literacy or better reporting of injuries [13]. These statistics highlight the need to improve conventional ACL injury prevention strategies while also suggesting that efforts should be specifically targeted toward athletes at most risk [14]. This is particularly important for sports and exercise scientists who are expected to prescribe individualised training plans supported by high-quality evidence. However, existing ACL injury prevention guidelines and review articles do not provide recommendations on how to create personalised and targeted training strategies [15–17].

The goal of this position statement is to provide practitioners with contemporary, evidence-based guidance for prescribing exercise to reduce the risk of ACL injury. Specifically, we aim to: (1) critically evaluate the effect of existing exercise interventions on ACL injury incidence, and (2) synthesise evidence on intrinsic risk factors and mechanistic pathways to guide targeted, evidence-informed training strategies for ACL injury prevention.

## Systematic Literature Searches

**Fig. 2:**
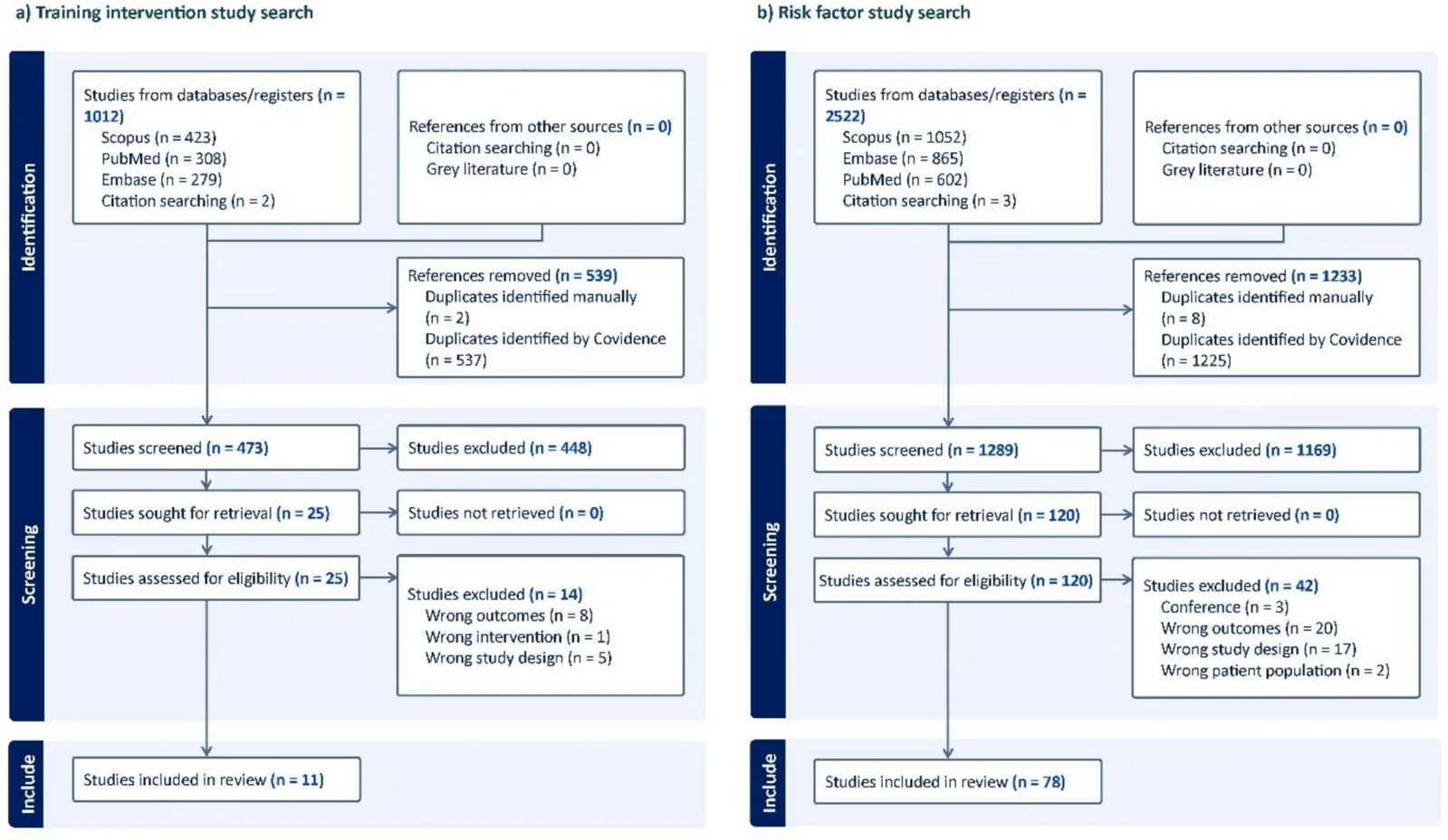
Preferred Reporting Items for Systematic Reviews and Meta-Analyses (PRISMA) flow diagram showing article identification, screening, eligibility and inclusion for two independent searches: **a** training intervention studies and **b** risk factor studies. Boxes report numbers of records retrieved from databases and citation searching, duplicates removed, records screened and excluded, full texts assessed with reasons for exclusion and final studies included in each review. Diagrams generated using Covidence

Systematic reviews of the literature were performed for (1) the effect of training interventions on ACL injury risk and (2) risk factors for non-contact ACL injury. Databases searched included Scopus, Embase and PubMed. The Preferred Reporting Items for Systematic reviews and Meta-Analyses (PRISMA) flow diagrams are presented in Fig. 2. A full list of search strategy keywords and a list of studies meeting the inclusion and exclusion criteria are presented in Online Resource 1. One author performed the literature search, screening and data extraction.

### Training Intervention Search and Selection Criteria

The PICOS framework was used to develop a systematic search strategy of keywords for active individuals (population), training programs (intervention), ACL injury (outcome) and randomised and non-randomised controlled trials and cohort/prospective studies (study design). The study inclusion criteria were (1) exercise-based training intervention, (2) control group consisting of no additional training or training as usual, (3) groups randomised or non-randomised by clusters or individuals, (4) reported ACL injury rates for each group and (5) published in a peer-reviewed journal. The study exclusion criteria were (1) no inclusion of a separate control group, (2) fewer than 5 ACL injuries (due to high risk of imprecise effects) and (3) conference posters or abstracts. Data extracted from each study included participant demographics, number of ACL injuries, statistical significance and relative injury risk. To evaluate the methodological quality of each study, Physiotherapy Evidence Database (PEDro) scores were extracted from nine previous systematic reviews and collated using a median value (Online Resource 1).

### Risk Factors Search and Selection Criteria

The PICOS framework was used to develop a systematic search strategy of keywords for active individuals (population), ACL injury or risk factor variables (outcome) and prospective or longitudinal cohort studies (study design). The study inclusion criteria were (1) analysed and reported one or more intrinsic risk factors (e.g. odds ratio, relative risk, group comparison), (2) non-contact ACL injury mechanism, (3) field and court sport population and (4) published in a peer-reviewed journal. The study exclusion criteria were (1) extrinsic risk factors only, (2) no univariable analysis of risk, (3) snow/water-based sports or police/military populations, (4) fewer than five ACL injuries and (5) conference posters or abstracts. Data extracted from each study included statistically significant intrinsic risk factors.

## Evidence for Reducing ACL Injury Risk

### Effectiveness of Exercise-Based Programs

**Fig. 3:**
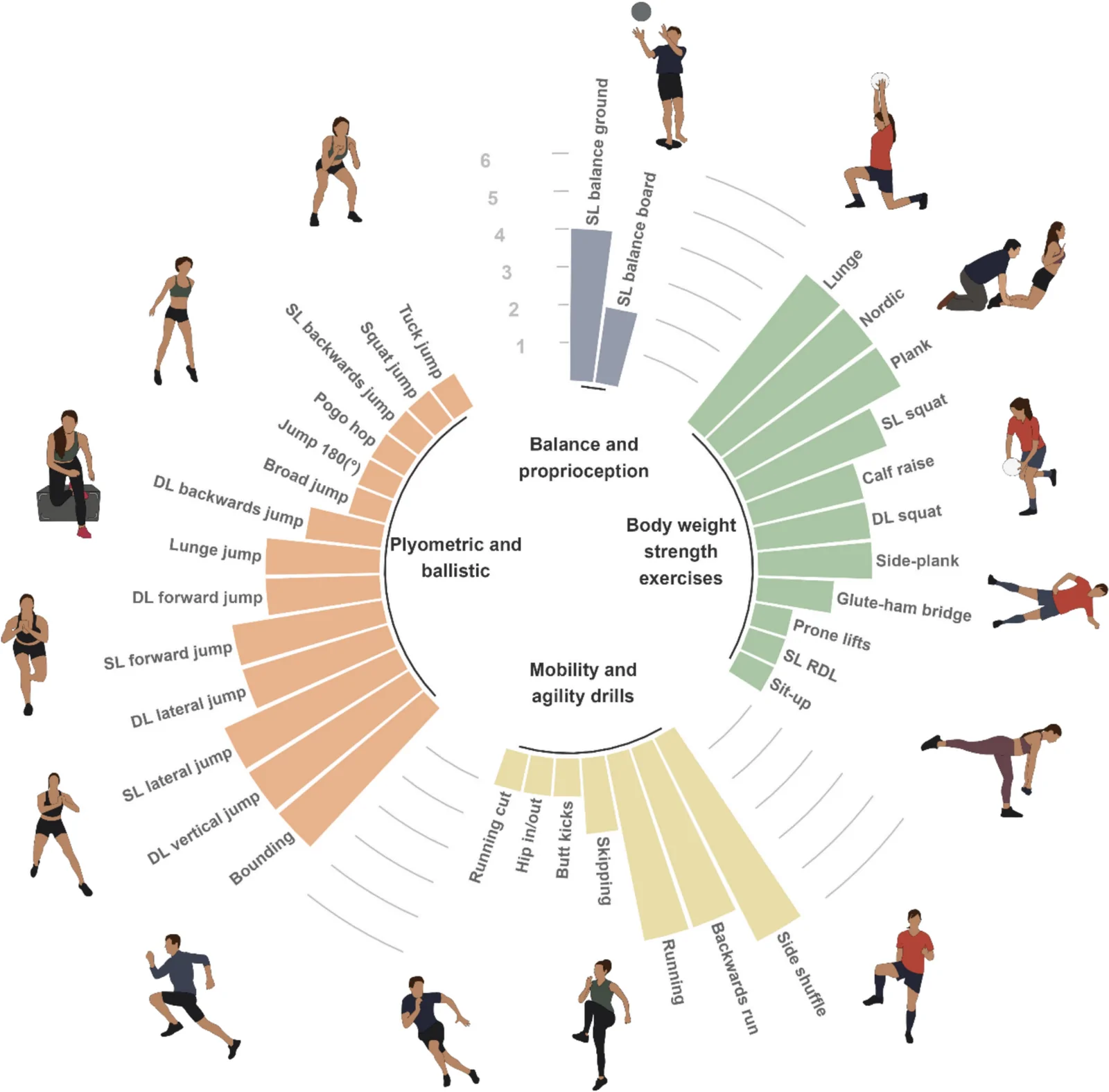
Exercises included in training interventions with demonstrated effectiveness in reducing ACL injury risk (*n* = 10 studies indicated by Table 1*Effectiveness* column, regardless of statistical significance). Bars represent the number of studies that included each exercise. DL, double-leg; SL, single-leg

Current guidelines and consensus statements based on controlled trials recommend exercise-based training programs (commonly termed ‘neuromuscular’ training) to reduce ACL injury risk [15, 18]. Meta-analyses, which combine results from all available studies, estimate that multi-component exercise programs reduce ACL injury risk by approximately 60% in female soccer, basketball or handball players aged 14–18 years [19, 20]. Table 1 provides a summary of all previous studies assessing the effectiveness of exercise-based training interventions for reducing ACL injury risk. In the largest cluster randomised controlled trial (RCT) performed to date, 121 of 230 Swedish female adolescent football teams were randomly assigned to a 15-min neuromuscular warm-up (‘Knee control’ program) consisting of body weight strengthening exercises and jump-landing drills (Fig. 3) completed twice per week during the season [21]. The intervention reduced ACL injury risk by 64%; however, this estimate was based on 7 injuries in 2479 players from the intervention group and 14 injuries in 2085 players from the control group, representing a relatively low overall injury rate compared with other sports [12]. Other RCTs focusing on injury prevention in female soccer, including the ‘Prevent Injury and Enhance Performance (PEP)’ program [22] and ‘FIFA 11’ [23], have shown 70% and 29% reductions in ACL injury risk, respectively (Table 1). These structured warm-up programs include dynamic running, stretching, strength, balance and plyometric exercises (Fig. 3). However, these findings were not statistically significant, partly owing to the small number of ACL injuries observed. Based on a systematic search of the literature, only one cluster RCT has been performed in males, showing a 76% reduction in ACL injury risk in college-level male soccer players with the ‘FIFA 11 + ’ program [24]. Additional non-randomised cohort studies have reported varying results for the effectiveness of proprioception and balance training [25, 26] and other multi-component exercise programs on ACL injury rates in female athletes [27–30] (Table 1).

**Table 1:** Summary of studies assessing the effectiveness of exercise-based training on ACL injury risk, including population, sport, age, competition level and study design characteristics based on a systematic search of the literature (Online Resource 1)

Program name^a^	Population				Number of participants (number of ACL injuries)		Results		Study quality	
	Sex	Sport	Age (years)	Level	Exercise	Control	Statistical significance	Effectiveness^b^	Design	PEDro (/10)^c^
Proprioceptive Training [25]	N/A	Soccer	N/A	Elite	300 (10)	300 (70)	Significant	**↓87% reduction** in all ACL injury risk	Non-randomised cohort study	3
Prevent Injury and Enhance Performance (PEP) [31]	Female	Soccer	14–18	Recreational	1885 (6)	3818 (67)	Significant	**↓82% reduction** in all ACL injury risk	Non-randomised cohort study	3
FIFA 11 + [24]	Male	Soccer	20–22	College	675 (3)	850 (16)	Significant	**↓76% reduction** in all ACL injury risk	Cluster randomised controlled trial	6.5
Knäkontroll (Knee control) [21]	Female	Soccer	13–17	Recreational	2479 (7)	2085 (14)	Significant	**↓64% reduction** in all ACL injury risk	Cluster randomised controlled trial	7
HarmoKnee [30]	Female	Soccer	13–19	Recreational	777 (0)	729 (5)	Non-significant	**↓100% reduction** in all ACL injury risk	Non-randomised cohort study	4
Proprioception and Jump Exercises [28]	Female	Handball	N/A	Recreational Elite	134 (1)	142 (5)	Non-significant	**↓80% reduction** in all ACL injury risk	Cluster non-randomised trial	2
‘Neuromuscular’ warm-up [32]	Female	Soccer, basketball	14–18	High school	485 (2)	370 (6)	Non-significant	**↓73% reduction** in all ACL injury risk	Cluster randomised controlled trial	6
Prevent Injury and Enhance Performance (PEP) [22]	Female	Soccer	18–22	College	583 (7)	852 (18)	Non-significant	**↓41% reduction** in all ACL injury risk	Cluster randomised controlled trial	4
Frappier Acceleration Training Program [27]	Female	Soccer	14–18	High school	42 (1)	258 (8)	Non-significant	**↓23% reduction** in all ACL injury risk	Randomised controlled trial	5
FIFA 11 [23]	Female	Soccer	13–17	Recreational	1073 (4)	947 (5)	Non-significant	**↓20% reduction** in all ACL injury risk	Cluster randomised controlled trial	7
Knee Ligament Injury Prevention (KLIP) Program [40]	Female	Soccer, basketball	14–18	High school	577 (3)	862 (3)	Non-significant	↑**114% increase** in non-contact ACL injury risk	Non-randomised cohort study	2
Balance Training [26]	Female	Soccer	15–26	Recreational	62 (4)	78 (1)	Non-significant	↑**277% increase** in non-contact ACL injury risk	Cluster randomised controlled trial	4

ACL, anterior cruciate ligament; N/A, not available (information not reported)

^a^Studies were included if they assessed an exercise-based training intervention, with a control group randomised or non-randomised by clusters or individuals, and reported more than a total of five ACL injuries

^b^Where not reported, relative risk reduction was calculated: (intervention injury rate – control injury rate)/control injury rate*100, using injury incidence per training or game exposure when possible. Elite includes players reported as professional, semi-professional or competing in a national-level competition. Age based on minimum to maximum range or approximated conservatively using mean ± 1 standard deviation

^c^PEDro scores based on median rating provided by previous systematic reviews [41–49]

When evaluating the strength of the current evidence, it is important to consider the individual studies. The overall effectiveness of multi-component programs is based on meta-analyses of a relatively small number of studies that are primarily at high risk of bias due to non-randomised or cohort study designs, low injury numbers (e.g. 50% of studies with less than 10 injuries) that may inflate relative risk and limited control of confounding factors (e.g. exposure, clustering, recruitment). Programs also range widely from a single exercise component to four or more components (Fig. 3). Further, there is limited evidence that these programs reduce ACL injury risk in males and elite athletes, and no evidence for many high-risk field- and court-based sports (e.g. Australian Rules Football, netball and rugby).

### Components of Exercise-Based Programs

Effective training programs commonly include plyometric and ballistic exercises (9/10 studies), body weight strength exercises (8/10 studies), balance and proprioception exercises (7/10 studies) and mobility and agility drills (7/10 studies). These exercises are designed to be included in place of the team’s regular warm-up, with minimal equipment, taking 10–30 min in total to complete, and performed 2–3 times per week (Table 2). Plyometric and ballistic movements (e.g. jumping, bounding, hopping and landing) are performed in multiple directions (vertical, lateral and forward) with maximal or near-maximal effort. Emphasis is placed on proper alignment of feet, knees and hips, and participants are encouraged to land with greater knee and hip flexion (i.e. ‘soft’ landings) [22, 23, 30–32]. Jump-landing drills can be progressed in intensity by changing from double-leg to single-leg movements and reducing time in contact with the ground. Strengthening exercises are primarily performed with bodyweight only, and commonly include lunges, Nordic hamstring curls, prone and side-planks, squats, calf raises and glute-ham bridges [21, 23, 32]. Balance or proprioception exercises alone show promise for reducing ACL injury risk both as a single component of training [25, 28], and when included as part of a multi-component program [21, 23]. Balance exercises are often performed on unstable surfaces such as wobble boards, foam pads and air-filled cushions with advancing complexity by introducing secondary tasks such as throwing and catching a ball or perturbations from another player [25, 28, 29]. Mobility and agility drills such as running and side-shuffling are also included in most ACL injury prevention programs, particularly those that are intended to replace the team’s normal warm-up [22, 31, 32]. A limited number of prevention programs also include stretching exercises for the calves, hamstrings, quadriceps and hip muscles [22, 31].

**Table 2:** American College of Sports Medicine Frequency, intensity, time, type, volume and progression (FITT-VP) principles for exercise prescription to reduce anterior cruciate ligament injury risk

Modality (included in effective studies)^a^	Frequency	Intensity and progression	Time and volume	Type
Plyometric and ballistic (9/10)	Multiple days per week ≥ 2–3 days/week Pre-season and in-season Before training and games Replace or integrate with warm-up	Jumps performed with moderate-to-high levels of effort Progress from double to single leg Increase intensity by decreasing ground contact time Add an external target or hurdle to encourage maximal effort Focus on movement quality: ‘Straight alignment hip-knee-foot’ ‘Low centre of gravity’ ‘Lightly flexed knees’ ‘Soft and controlled landings’	8–15 reps per exercise 1–3 sets per exercise 5 min total	Bounding Multi-directional hop Lunge jump Broad jump Pogo jump Squat jump Tuck jump
Body weight strength exercises (8/10)		Progress body weight exercises from double to single leg Add external resistance when able to perform > 10–15 repetitions Focus on movement quality ‘Straight alignment hip-knee-foot’ ‘Straight torso’	8–15 reps per exercise 1–3 sets per exercise Isometric exercise > 15–30 s duration 5 min total	Lunge Nordic hamstring curl Prone plank Side plank Squat Calf raises Glute-ham bridge
Balance and proprioception (7/10)		Progress balance exercise from more stable surfaces to less stable surfaces Include secondary tasks such as catching, throwing, or perturbations Close eyes to increase reliance on proprioception	Balance exercise > 15–30 s duration 5 min total	Single-leg balance on ground Single-leg balance on foam or wobble board Jump-landing on foam or wobble board Include catch/throwing task or eyes closed
Mobility and agility drills (7/10)		Light-to-moderate intensity (< 5/10 RPE) and long duration for warm-up benefits High intensity (> 7/10 RPE) and short duration (< 30 s) for neural benefits	5–15 min total depending on the sport	Side shuffle Forwards or backwards running Skipping Dynamic stretches

RPE, rate of perceived exertion

^a^Effective studies based on a reduction in ACL injury risk in Table 1

### Limitations of Current Injury Prevention Programs

Adherence to current injury prevention programs can be relatively low, even when performed under clinical trial conditions (mean of 66 ± 21%, *n* = 19 studies) [33]. Given that multi-component training programs include many different training modalities and exercises (Fig. 3), it is unclear which components are responsible for reducing ACL injury risk or the mechanisms underpinning their effectiveness [34]. Risk reduction strategies can still be successful despite a lack of knowledge about the exact mechanisms by which they work. This is often the case in many areas of sports medicine, where successful interventions have been developed using available evidence, expert opinion, clinical experience, anecdotes or trial and error effects [21, 35]. However, these approaches may result in poor use of time, higher costs and heterogeneous responses among individuals. Without understanding the key adaptations or exercises responsible for ACL injury risk reduction, there is limited scope for exercise professionals to create personalised training programs or for researchers to establish more targeted and effective prevention approaches [36]. Further, injury prevention programs may include redundant exercises that unnecessarily increase training duration, which is a key barrier to implementation [37]. Notably, most contemporary ACL injury prevention training programs [38] consist of similar exercise strategies to those proposed 30 years ago [39].

## Aetiology of ACL Injury

### Injury Mechanism

**Fig. 4:**
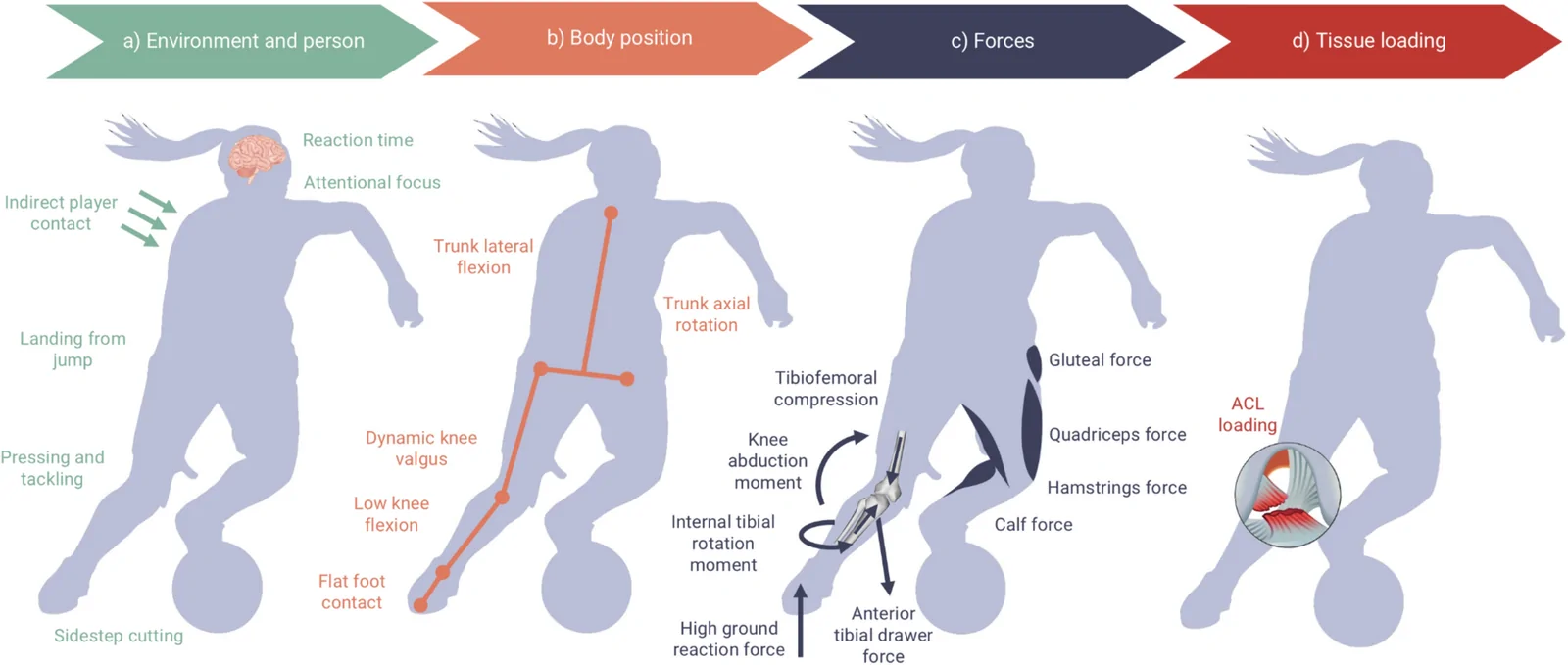
The mechanistic pathway of anterior cruciate ligament (ACL) injury: a) environmental factors, playing situation, and player behaviour, b) whole-body posture and movements, c) external forces, joint moments, and muscle forces, and d) ACL tissue loading. Factors included in the diagram are based on evidence from video analyses [51, 56–58], musculoskeletal models [68, 71] and cadaveric experiments [53, 67, 72–76]

Understanding the mechanistic pathways that lead to ACL injury is a critical step in designing more targeted and potentially more effective prevention training [36, 50]. Ultimately, ACL rupture is caused by tissue loading that exceeds the current tissue strength, which varies as a function of age, sex, training and injury. The mechanistic pathway leading to excessive tissue loading can be studied from distal features, such as describing the inciting event, playing situation, player/opponent behaviour and observed movement, through to more proximal features such as the joint and tissue-level biomechanics (Fig. 4) [36]. Evidence for ACL injury mechanisms is typically established from four different study designs with various strengths and limitations: (1) video analysis at the estimated time of injury that captures the surrounding events and estimates of body postures, but not the mechanics responsible for the injury [51]; (2) in vivo laboratory studies that allow measurement of joint loads (moments and forces) during different experimental conditions, but without any ACL injuries occurring [52]; (3) in vitro laboratory studies that allow controlled forces to be applied until tissue failure, but within human cadavers under simulated conditions [53]; and (4) in silico experiments that use computer simulations to estimate forces within the body based on a simplified representation of human biology [54, 55].

On the basis of video analysis research, ACL injury most commonly occurs without contact with other players during change of direction manoeuvres when trying to evade or defend an opposition player, when lunging to make a tackle, or landing from a jump (Fig. 4a) [51, 56, 57]. Many injury scenarios involve ‘indirect’ contact from another player or object (but the force is not applied directly to the knee) that perturbs body movement immediately before the injury [58]. Female athletes are more likely than male athletes to incur non-contact or indirect contact ACL injuries, particularly at the elite-level [59]. Injuries that occur without contact are theoretically more preventable (i.e., via athlete-based interventions) than contact-induced injuries.

Given that ACL injuries often occur during movements that have been performed safely countless times, it is conceivable that neurocognitive ‘errors’ between movement intent and coordination of muscles are implicated in the mechanism of ACL injury [60]. Limited time to plan and execute movements during reactive tasks, together with split attentional focus characterize the events surrounding most ACL injuries. For example, unanticipated sidestep cutting increases frontal and transverse plane external knee moments compared with pre-planned sidestep cutting [61], reacting to sports-like visual stimuli increases trunk lateral flexion [62] and performing tasks with divided attention decreases knee flexion and increases knee valgus [63]. ACL rupture is estimated to occur less than 50 ms after initial foot contact with the ground [64], which is an insufficient amount of time for voluntary or even reflexive activation of muscles [65].

At the time of injury, body postures typically include near full knee extension, dynamic knee valgus due to combined knee abduction, hip adduction and hip internal rotation, ankle eversion, a flat foot position, lateral trunk flexion over the stance limb and trunk rotation away from the stance limb (Fig. 4b) [51]. Owing to this body posture and the high ground reaction forces produced during a forceful step, the tibiofemoral joint is exposed to high compressive forces, and large knee abduction and tibial internal rotation moments (Fig. 4c) [66]. Compression of the tibiofemoral joint alone results in a ‘pivot-shift’ mechanism generating an anterior tibial drawer force, as well as knee abduction and internal tibial rotation moments due to the posterior sloping tibia which is steeper in medial versus lateral compartments [67]. It is the combination of these forces and moments that have been shown to produce maximum ACL strain [53]. Muscle forces may also increase or decrease loads on the ACL depending on the muscle line of action relative to the knee joint centre (i.e. moment arm), as well as joint position [68]. For example, during landing and side-step cutting, the quadriceps and gastrocnemius are primarily responsible for increasing anterior drawer force, while the hamstrings and soleus produce a posterior tibial drawer force, thereby supporting the ACL [69, 70]. The multi-planar forces and moments are then transferred to the ACL to produce a resultant force on the tissue through complex non-linear dynamics (Fig. 4d) [68].

### Intrinsic Risk Factors

To date, numerous ACL injury risk factors have been proposed. Based on a systematic review of prospective studies (Table 3), prior ACL injury is the most consistently identified intrinsic risk factor for future ACL injury, reflected by the high rates of re-injury following ACL reconstruction surgery [77–79]. Additionally, there is strong evidence that several morphological factors are associated with ACL injury risk, including smaller ACL size [80, 81], greater tibial posterior slope [81, 82] and a narrower intercondylar notch [83–85]; however, these factors are largely non-modifiable. Many of these morphological features are also hereditary, which may partly explain the elevated risk of ACL injury in those with a family history of ACL injury or with specific gene variants [86, 87].

**Table 3:** Summary of proposed intrinsic risk factors for first-time ACL injury in sporting populations based on a systematic search of the literature (Online Resource 1)

Category	Proposed intrinsic risk factor	Reference ^a^	Potential mechanistic rationale^b^
Morphology	↑ Body weight	[90, 114]	*Directly* increases ground reaction forces and external knee joint loads
	**↓** ACL size	[80, 81]	*Directly* reduces ACL ultimate strength
	↑ Generalised joint laxity	[115]	*Associated with* decreased ACL stiffness and ultimate strength
	↑ Anterior tibial drawer laxity	[115, 116]	*Associated with* decreased ACL stiffness and ultimate strength
	↑ Passive knee extension	[117]	*Associated with* decreased ACL stiffness and ultimate strength
	↑ Posterior tibial slope	[81, 82] [118–124]	*Directly* increases anterior tibial drawer force with tibiofemoral compression
	**↓** Intercondylar notch width	[83–85, 123, 125–127]	*Directly* increases the risk of impingement *Associated with* smaller ACL size and strength *Associated with* ligament moment arms
	**↓** Femoral anteversion	[114]	*Directly* increases external knee loads
Biomechanical	↑ Dynamic knee valgus/abduction	[14, 77, 88, 98, 99, 101]	*Directly* increases knee valgus loading and ACL strain
	**↓** Knee flexion	[14, 98, 128]	*Directly* increases quadriceps tibial anterior drawer force *Directly* reduces hamstrings tibial posterior drawer force *Directly* increases ground reaction forces and external knee loads
	↑ Knee internal rotation	[95]	*Directly* increases knee internal rotation loading and ACL strain
	**↓** Hip flexion	[95, 100]	*Directly* increases ground reaction forces and external knee loads *Associated with* trunk angle and the external lever arm between the trunk and the knee
	↑ Lateral trunk flexion	[77, 88] [129]	*Directly* increases external lever arm between trunk and knee joint and greater knee valgus loading
	↑ Lateral pelvic hike	[130]	*Associated with* trunk angle and external lever arm between the trunk and the knee
	↑ Jump force and impulse	[77, 131]	*Associated with* greater quadriceps force production and anterior tibial drawer force
	↑ Vertical ground reaction force	[14, 128]	*Directly* increases external knee joint loads *Directly* increases tibiofemoral compression force
	↑ Knee abduction moment	[14, 132]	*Directly* increases knee valgus loading and ACL strain
	↑ Knee flexion moment	[100]	*Directly* increases quadriceps muscle force, anterior tibial drawer force, and ACL strain
	↑ Hip flexion moment	[14]	*Associated with* increased GRF
	↑ Landing Error Score System	[133]	*Associated with* greater external knee joint loading
	↑ Double leg standing, locus length	[90, 134]	*Associated with* decreased joint proprioception and control
	↑ Time to stabilisation during backwards jumping	[135]	*Associated with* decreased joint proprioception and control
Neuromuscular	**↓** Isokinetic knee flexion	[136]	*Directly* decreases posterior tibial drawer force
	**↓** Isometric hip adduction:abduction ratio	[77]	*Associated with* reduced control of frontal plane hip movements
	**↓**/**↑**Isometric hip abduction	[89, 90]	*Associated with* reduced control of frontal plane hip movements
	**↓** Isometric hip external rotation	[95]	*Associated with* greater knee valgus loading
	**↓** Isokinetic hamstring:quadriceps ratio	[91]	*Directly* decreases posterior tibial drawer force and/or greater anterior tibial drawer force
	**↓** Pre-activation of semitendinosus	[94, 95]	*Directly* decreases posterior tibial drawer force
	↑ Pre-activation of vastus lateralis	[94]	*Directly* increases anterior tibial drawer force
Psychological	↑ Psychological competitive ability	[137]	*Associated with* risk-taking behaviours
Hormonal	Follicular phase of the menstrual cycle	[138–140]	*Directly* increases ligament laxity and may reduce ACL strength
	↑ Serum relaxin concentration	[141]	*Directly* increases ligament laxity and may reduce ACL strength
Injury history	ACL injury history	[77–79, 115, 142–144]	*Directly* reduces ligament strength *Associated with* reduced joint proprioception *Associated with* reduced muscle size, strength, and activation *Associated with* movement apprehension and limb shielding
	Family ACL injury history	[86, 87] [115, 145, 146]	*Associated with* ligament size and strength *Associated with* ligament moment arms *Associated with* behavioural and psychological attributes
	Concussion history	[147]	*Associated with* impaired reaction time, attentional capacity, and decision-making
Genetic	Gene variants	[148–150] [151–154]	*Directly* influences ligament size and strength *Directly* influences muscle size, strength, and activation *Directly* influences psychological factors
	Sex (biological)	[12]	*Associated with* a smaller ligament size and strength *Associated with* smaller ligament moment arms *Associated with* hormone concentration and ligament laxity *Associated with* muscle size and strength

^a^Studies were included if they reported a significant univariable intrinsic risk factor for a field or court sport population based on a prospective study design with more than five ACL injuries. Additional case–control or retrospective studies were considered for variables that are non-modifiable or able to be accurately retrieved from records

^b^Note this list is not intended to provide every possible rationale. ACL, anterior cruciate ligament

A range of kinematic and kinetic variables have been proposed as ACL injury risk factors (Table 3). Among these, greater knee abduction angles (i.e. measured with three-dimensional motion capture systems) or dynamic knee valgus (i.e. measured with two-dimensional surrogates), low knee flexion, lateral trunk flexion and high ground reaction forces appear to be the most commonly identified biomechanical risk factors for ACL injury [14, 77, 88]. Importantly, these movement characteristics are associated with increased external knee joint loads [52].

Several neuromuscular factors, particularly those related to lower limb strength and muscle activity, have also shown associations with subsequent ACL injury. A limited number of prospective studies have reported associations between lower hip or thigh muscle strength and subsequent ACL injury [89–91]; however, current evidence does not support the superiority of any specific strength testing modalities (e.g. isokinetic or isometric) or contraction mode (e.g. concentric or eccentric) for identifying injury risk. In the absence of direct evidence linking strength measures to injury risk, it might be reasonable to prioritise assessment of muscles that have the greatest potential to increase or decrease ACL loads, such as the knee extensors and flexors, hip abductors and extensors, hip external rotators and ankle plantar flexors [92, 93]. Limited evidence also suggests that greater vastus lateralis and semitendinosus muscle activity before ground contact (pre-activation) is associated with lower ACL injury risk [94, 95]. Co-activation of the quadriceps and hamstrings helps to stabilise the knee against valgus/varus knee loading [96], while muscle pre-activation helps stabilise against impact forces that occur too quickly for reactive sensorimotor muscle activation [65]. Many other factors may contribute to ACL injury risk but have not been examined in prospective studies and are therefore not included in this review (e.g. muscle–tendon unit stiffness, fatigue and rate of force development).

### Assessing Injury Risk

For biomechanical assessments of injury risk, the choice of device and movement task is important to ensure measurement accuracy and ecological validity (i.e. results reflect biomechanics that occur in sports). Based on motor control principles, it is likely important that the task is in some way representative of the movement characteristics related to the ACL injury mechanism (e.g. forceful accelerations with limited ground contact time, single-leg stance or multidirectional deceleration and change of direction) [97] (Fig. 4). Based on these criteria and considering prospective evidence for assessing injury risk, tasks such as bilateral drop vertical jumps [14, 98–100], single-leg drop vertical jumps [88, 101], single-leg triple vertical hops [77] and running sidestep cuts should be considered [95]. Given that movement is highly dependent on task constraints and neurocognitive processes, the development of more sport-specific tasks with game-like stimuli may improve the ecological validity of biomechanical testing [102]. Further, the utility of assessing biomechanics under fatigued conditions may also be relevant for representing game conditions. However, it is hypothesised that fatigue may also have a protective effect by reducing movement velocities and muscle force production that could contribute to reducing ACL loading [103]. To assess biomechanics, marker-based optical camera systems provide the greatest accuracy and reliability [104]. However, markerless pose estimation from video cameras, depth cameras or inertial measurement units are capable of recording motion to within approximately 5 to 10° compared with marker-based motion capture depending on the joint and plane. Examples of current devices include Theia multiple video cameras [105], OpenCap dual smartphones [106], Microsoft Azure depth camera [107] and Xsens IMUs [108].

In sports medicine, screening is commonly performed to identify athletes at risk for injury. This typically involves administering a clinical test(s) and separating individuals into low- or high-risk based on a cut-off value proposed in the literature [109]. It should be noted that optimal cut-off values from the literature are often highly specific to a study’s sample without measures taken to reduce model overfitting and do not generalise to newly collected data or to different cohorts [110, 111]. Although injury is binary (injured/not injured), injury risk is the possibility of an injury occurring from 0–100% certainty [112]. As such, quantifying injury risk on a continuous scale using probability is most appropriate. Based upon estimated injury risk, it is then up to the practitioner and/or individual to decide on an acceptable level of risk as to whether intervention is required. Given that injury is multifactorial, it is unlikely that any single test or variable will accurately identify injury risk. Instead, a combination of key predictors should be used in a multivariable statistical model. However, the availability of data to develop such a prediction model is currently a major limiting factor for practitioners wishing to implement accurate ACL injury risk assessments.

It is important to be aware that, without carefully controlling for all confounding variables, risk factors derived from observational studies represent statistical associations and not causal effects [113]. For example, strength may be statistically correlated with lower injury risk because both are influenced by an athlete’s training history, rather than strength itself directly reducing injury risk (i.e., confounding). As such, intervening to change a risk factor may not necessarily impact injury risk [113]. Further, accounting for all possible confounding variables in an observational study is often impractical, and therefore, unbiased causal risk factors cannot be established with this study design alone. In the absence of direct evidence for causal risk factors, it may be useful to consider whether there is a strong mechanistic rationale (i.e. a cause-and-effect relationship) between a proposed risk factor and injury occurrence (See Table 3) [55]. Nevertheless, appropriately designed prospective cohort studies can provide valuable information to inform injury prevention training.

Based on current evidence, physical testing cannot yet be recommended for the purpose of identifying actionable metrics to reduce ACL injury risk. Practitioners may still use testing to identify asymmetries or deficits relative to expected benchmarks (particularly following injury); however, it remains unclear whether correcting such deficits reduces the risk of first-time or subsequent ACL injury.

### Consideration of the Effects of Sex and Gender on ACL Injury Risk

There are many contributing factors to ACL injury that are influenced by sex and/or gender. Sex-related factors refer to biological characteristics (e.g. anatomy) and gender-related factors refer to the roles, identities and structures adopted by the sexes and influenced by society (e.g. the training and competition environment) [155]. For example, on average, females have less ACL volume, smaller ligament moment arms and increased ligament laxity, resulting in lower tolerance to knee loading compared with males [156–158]. However, on average, females also have shorter limb lengths and less total body mass than males, thus reducing the net loading of joints. Social and environmental factors also contribute to ACL injury risk. Social norms and available opportunities may impact the type/quantity of training, coaching and support received by girls and women over their lifespan [159]. Social factors may therefore contribute to the physical (e.g. lower muscle mass and strength in females compared with males) and biomechanical (e.g. greater knee valgus in females during landing) sex differences that have been frequently reported [160, 161]. Without research that quantifies the numerous contributing factors to ACL injury, it is difficult to establish the relative contribution of sex and/or gender-related factors in explaining differences in ACL injury rates. To achieve significant reductions in ACL injury risk, it is likely important to establish both social changes and implement evidence-based training strategies. Nevertheless, invoking positive changes to social norms, training and competition environments alone may have benefits beyond ACL injury prevention (e.g. equality of opportunity or performance benefits).

## Effect of Exercise Interventions on ACL Injury Mechanisms and Risk Factors

Understanding the impact of exercise interventions on ACL injury mechanisms and risk factors is an important step towards developing targeted and individualised prevention strategies. To date, no RCTs have directly linked specific exercises to a reduction in ACL injury incidence; rather, existing research has evaluated multi-component programs incorporating a range of training modalities. By examining the effect of exercise interventions on modifiable factors – such as biomechanics, strength, balance and morphology – practitioners can make more informed decisions when designing injury prevention programs. This approach offers a practical and evidence-informed strategy for mitigating ACL injury risk, particularly in high-risk populations, by addressing the underlying contributors to injury.

### Biomechanical Factors

Avoiding body postures that increase ACL loading may help reduce the risk of injury [162]. Movement training that focuses on increasing knee flexion, reducing knee valgus, narrowing stance width and maintaining a neutral trunk position during change of direction has been shown to reduce external knee joint loading [163, 164]. In a meta-analysis of the effect of ACL injury prevention training on kinematic and kinetic variables during dynamic tasks, small increases in knee flexion angle (0.8–5.5°) were the only consistent change observed [165]. Small increases in knee flexion may partly explain the mechanism by which multi-component and warm-up training programs reduce ACL injury risk. However, existing ACL injury prevention programs do not consistently alter external knee joint loading or other kinematic variables during change of direction, landing and hopping [165, 166]. Nevertheless, individual studies have shown positive benefits of change of direction exercises [167] and dynamic or isometric strength training [168] for reducing knee valgus angles and moments. Further, changing an individual’s movement patterns to reduce knee loads in training may not always transfer to in-game situations and may have detrimental effects on performance [169]. Successfully modifying in-game biomechanics to reduce ACL injury risk may therefore require more deliberate and targeted training approaches. Prevention strategies may consider incorporating more ‘neurocognitive challenges’ such as (1) reduced reaction time during reactive or unanticipated tasks, (2) dual-tasking and decision making, (3) learning to ignore ball fakes and irrelevant external information during a task (selective attention) or (4) adding heightened emotional/competitive pressures to training tasks [65].

Exercise combined with either visual or verbal feedback may be an effective strategy for modifying biomechanical risk factors. Preliminary studies combining real-time augmented visual feedback (e.g. overlayed movement targets) with strength, plyometric and balance training have shown improved brain activation patterns and favourable biomechanical changes during landing [170]. Other studies have shown reductions in external knee joint loads following 6 weeks of side-step cutting training involving video feedback [163], 6 weeks of exercises with mirror feedback [171] and 8 weeks of strength, plyometric and conditioning training with video feedback [172]. For verbal feedback, instructions with an external focus of attention (i.e., focusing on an outcome outside of the body) enhance motor learning compared to instructions with an internal focus [173]. Further, self-controlled timing of feedback has been shown to enhance improvements in kinematics during unanticipated sidestep cutting compared with scheduled feedback, indicating the benefit of feedback autonomy [174]. Psychological attributes may also interact with neurocognitive function, with more aggressive, motivated or competitive athletes likely to select high-risk movement strategies that place a greater load on the ACL [137].

### Neuromuscular Factors

Actively generated muscle forces can either assist or oppose the ACL in stabilising the knee joint [175]. When the knee is flexed, the main muscles capable of supporting the ACL via posterior tibial drawer force include the hamstrings, soleus, sartorius and gracilis [92]. The gluteus medius, gluteus maximus and piriformis make the greatest contribution to opposing knee abduction moments [70]. Muscle force-generating capacity is adaptable through resistance training [176], and specific exercises can elicit preferential hypertrophy of muscles that contribute favourably to ACL support. For example, 12 weeks of lengthened state eccentric knee flexion training resulted in greater growth of the biceps femoris long head and semimembranosus than the Nordic hamstring exercise, which promoted greater hypertrophy of biceps femoris short head and sartorius [177]. Additionally, seated calf raises can stimulate soleus hypertrophy while minimising gastrocnemius development [178].

However, maximum force-generating capacity alone may not be sufficient for ACL protection as ACL rupture occurs faster than voluntary muscle activation, highlighting the importance of pre-activation prior to ground contact [65]. Altering muscle activation patterns may be a viable solution; for example, a 12-month multi-component ACL injury prevention program significantly increased hamstring pre-landing muscle activity during a side-step manoeuvre [179]. However, it is important to consider that muscles have multi-planar moment arms and greater force production of a muscle may result in non-intuitive changes in loading at the knee or adjacent joints. As such, the neural control of muscles to support the ACL requires a careful balance and the optimal muscle recruitment strategy for a given individual is unclear.

Mechanical properties of muscle and tendon such as stiffness of the series and parallel elastic components are also important for modulating force output [180]. Therefore, increasing the muscle–tendon stiffness of supporting muscles via targeted training (e.g. high strain exercises) may help directly oppose ACL loading with lower amounts of muscle activation (e.g. hamstring stiffness) [181].

### Morphological Factors

The ultimate strength of the ACL is determined by its geometry and material properties. In animal models, 4–8 weeks of treadmill running has been shown to increase ACL cross-sectional area, stiffness, and ultimate strength in rats and dogs compared to no training or immobilisation [182–184]. In humans, the effect of exercise on ACL geometry has only been investigated in cross-sectional studies comparing cohorts with and without chronic habitual loading [185] or comparing limbs in sports involving unilateral loading [185, 186]. Compared with age-matched controls, weightlifters have been shown to have 55% greater ACL cross-sectional area [185]. In a study of figure skaters and springboard divers (i.e. sports that involve frequent unilateral loading), there was approximately a 5% difference in ACL cross-sectional area between limbs [186]. However, the retrospective nature of these studies makes it impossible to determine if the observed differences predated or occurred as a consequence of training. Future work is needed to determine the effect of targeted training interventions on ACL strength and, ultimately, injury risk.

## Conclusions and Recommendations

Recommendations are presented with the level of evidence based on study design and separated into two tiers. Tier 1 indicates population-level strategies with evidence for effectiveness in reducing ACL injury risk. Tier 2 indicates individualised and targeted strategies with evidence based on changing mechanisms/risk factors for ACL injury.

### Tier 1: Population-Level Strategies

Exercise-based interventions demonstrate strong potential for reducing ACL injury risk, particularly among young female athletes participating in cutting and landing sports (Table 4, Tier 1). There is strong evidence that structured neuromuscular or warm-up-based programs reduce ACL injury incidence by 60% when implemented at the population level. Evidence for potential effectiveness in males, elite athletes and sports beyond soccer, handball and basketball is emerging but remains limited. Neuromuscular or warm-up programs should therefore form the foundation of ACL injury prevention strategies. Interventions shown to be effective typically incorporate multiple exercise modalities, including strength training, balance, proprioception, plyometrics, mobility and agility exercises (Fig. 3). These programs are commonly performed 2–3 times per week for 15–20 min and can be integrated into regular training sessions, often replacing the traditional warm-up (Table 1). Within these programs, emphasis is placed on maintaining appropriate lower limb alignment, with athletes encouraged to adopt greater hip and knee flexion during landing tasks (i.e., soft landings). Strength training exercises are therefore most often performed with bodyweight only and without additional equipment.

**Table 4:** Summary of practical recommendations for reducing ACL injury risk with exercise-based strategies

Level of evidence	Evidence based on:	Study type/design
Level 1	Reducing injury risk	Meta-analysis of randomised controlled trials
Level 2	Reducing injury risk	A single randomised controlled trial
Level 3	Reducing injury risk	Non-randomised controlled trial, prospective, or longitudinal study
Level 4	Changing mechanism/risk factor	Meta-analysis of randomised controlled trials
Level 5	Changing mechanism/risk factor	A single randomised controlled trial
Level 6	Changing mechanism/risk factor	Non-randomised controlled trial, prospective or cross-sectional study
Adapted and expanded Oxford Centre for Evidence-Based Medicine 2011 Levels of Evidence		

Level of evidence	Tier 1: Population-level strategies (based on reducing injury risk)	Supporting references
Level 1	Young female athletes participating in soccer, basketball and handball should participate in a structured neuromuscular/warm-up program consisting of strengthening, balance, plyometrics, mobility and agility exercises	[19] [187]
Level 1	The effectiveness of neuromuscular/warm-up programs is enhanced when performed a minimum of two times per week with a duration of 20 min or more	[188]
Level 1	High compliance (> 66–88% of prescribed sessions) substantially increases the effectiveness of neuromuscular/warm-up programs compared with low compliance (< 66–88%)	[189] [190]
Level 2	Neuromuscular/warm-up programs may be effective at reducing ACL injury risk in male college soccer players	[24]
Level 2	Neuromuscular/warm-up programs may be adapted for specific sports; however, only if they contain the same exercises investigated by one of the effective randomised controlled trials. (e.g. Soccer: Australia Perform+ , AFL: Prep-to-Play, FootyFirst, Netball: The Knee Program)	[191] [31] [22] [21]

Level of evidence	Tier 2: Targeted intervention strategies (based on changing mechanism/risk factor)	Supporting references
Level 4	Multi-component training programs consisting of plyometric, strength or balance training may be effective at increasing knee flexion angle during landing and change of direction	[165]
Level 5	When instructing movement technique, the use of cues that encourage an external focus of attention, as opposed to an internal focus of attention, may increase knee flexion angle and decrease vertical ground reaction forces	[173]
Level 5	Quadriceps strength training may decrease knee valgus moment, internal rotation moments and dynamic knee valgus during single-leg landings	[192]
Level 6	To reduce external knee loading (i.e. joint moments), training should focus on limiting the following body postures during change of direction, deceleration and landing: low knee flexion, dynamic knee valgus, flat-foot ground contact, wide step width, lateral trunk flexion and trunk axial rotation	[193] [52] [194]
Level 6	To increase muscular support to the ACL, training should focus on increasing muscle force of the hamstrings (reduce anterior tibial drawer force), quadriceps and hamstring co-activation (reduce knee valgus moment), soleus (reduce anterior tibial drawer force), and gluteus medius and maximus (reduce knee valgus moment)	[92] [96]
Level 6	Change of direction drills with verbal or visual feedback may be effective at reducing external knee valgus moments during sidestep cutting	[163] [167]
Level 6	Gluteal and abdominal strength and motor control training may decrease dynamic knee valgus and lateral trunk flexion during single-leg squatting	[195] [196] [197]

Tier 2 strategies are suggested as adjunct interventions to Tier 1 strategies with demonstrated effectiveness

ACL, anterior cruciate ligament

### Tier 2: Targeted Strategies

Targeted interventions may be considered an adjunct to neuromuscular warm-up programs (Table 4, Tier 2). The objective of this training is to reduce the likelihood that ACL loading exceeds tissue tolerance by altering movement mechanics, increasing tissue strength or both. Evidence from biomechanical studies suggests that increasing knee flexion, reducing dynamic knee valgus, lateral trunk flexion and excessive step width can reduce external knee loading in high-risk tasks. Plyometric and strengthening exercises, as well as motor learning drills with visual or verbal feedback, including the use of external focus cues, have been shown to increase knee flexion and reduce ground reaction forces during landing. Technique-focused drills with video and/or coach feedback for sidestep cutting and landing may help to improve kinematic risk factors such as knee flexion, knee valgus and lateral trunk flexion. Implementing neurocognitive challenges, including reactive decision-making, dual-tasking, selective attention and competitive pressures, might also improve the transfer of training to game scenarios.

Targeted strengthening of muscles that support the ACL during high-risk tasks may be another important component. Gluteal, quadriceps and abdominal strengthening and motor control drills can reduce dynamic knee valgus, external knee moments and lateral trunk flexion. Coactivation of the quadriceps and hamstrings during weight acceptance further helps to stabilise the knee against varus/valgus knee loading. Progressive overload may be achieved by increasing the resistance/weight lifted, as well as manipulating task complexity, progressing from bilateral to unilateral exercises or increasing movement velocity.

Importantly, Tier 2 recommendations have not been shown to reduce ACL injury incidence and should therefore be prescribed selectively rather than as population-wide prevention strategies. At present, no externally validated screening test can accurately classify an individual’s risk of future ACL injury. Tier 2 recommendations should therefore be applied at the discretion of the practitioner, rather than following pre-determined rules (e.g. univariable cut-offs for specific risk factors). In practice, targeted interventions might be considered for athletes with greater exposure to high-risk tasks (e.g. owing to sport or playing position-specific demands) or a history of ACL injury (given the elevated rates of re-injury). Collectively, this tiered framework allows clinicians to design best-practice ACL injury prevention strategies that are aligned with both the strength and limitations of the current evidence base, prioritising population-level risk reduction while allowing selective individualisation on the basis of a mechanistic rationale.

### Recommendations for Future Research

Future RCTs are required to determine whether existing neuromuscular and warm-up programs reduce ACL injury risk in different cohorts (by age, gender, sport and competitive level). Further work is required to identify the essential components of existing programs that reduce ACL injury risk. Research should examine individualised training strategies to reduce ACL loading (e.g. improving body posture and muscular support) and/or increase ACL tissue strength (e.g. size and stiffness). Researchers should be cautious of using biomechanical proxy measures to infer ACL loading and injury risk (e.g. external ground reaction forces or external joint moments), as these may not accurately reflect ligament loading. The development and adoption of new technologies capable of quantifying ACL loading in the field or clinic may enable significant advancements. When investigating ACL injury risk factors, it is essential to distinguish between prediction and causation, ensuring appropriate measurement and adjustment for confounding factors. Additionally, future work is needed to externally validate screening batteries and establish clinically meaningful thresholds associated with ACL injury risk. Finally, future research should explore strategies to improve the adoption and adherence to ACL injury prevention training by co-designing strategies with coaches, practitioners and athletes, and by identifying the barriers to effective implementation across diverse populations (Table 5).

**Table 5:** Summary of recommendations for future research on reducing ACL injury risk with exercise-based strategies

Future research
**Establish the effectiveness of current and new ACL injury prevention strategies:** – More studies are required to understand the effectiveness of current ACL injury prevention programs in elite athletes, high-risk sports (e.g. Australian Rules Football, netball, and rugby) and males – New proposed strategies need to be assessed using high-quality randomised controlled studies – Examine whether training interventions targeting known or proposed risk factors can reduce ACL injury rates – Investigate whether combining injury screening with personalised training can improve the effectiveness of ACL injury prevention
**Identify causal risk factors for ACL injury:** – Researchers should be aware of whether their research questions are based on prediction or causation – Care should be taken not to interpret associations as causal relationships – Greater efforts should be made to measure and adjust for confounding factors during analysis – Selecting appropriate study designs to understand injury risk and causation – Quantifying the size of the effect is important to establish the importance of causal risk factors and potential impact on injury risk
**Develop technology to quantify ACL loading in training and game environments:** – External proxy measures of ACL loading are unable to fully quantify the stresses and strains experienced by the ACL tissue – Computer vision or wearable devices combined with a neuromusculoskeletal model will enable individualised ACL loading to be estimated and may be used to inform injury prevention strategies
**Determine the most effective exercise strategies for reducing ACL injury risk:** – Explore the mechanisms underpinning the effectiveness of current programs – Identify the optimal training strategies for targeting causal risk factors – Determine the optimal strategies for different groups or individuals
**Identify strategies to improve the adoption of and adherence to ACL injury prevention training:** – Co-design strategies with key stakeholders and end-users, including coaches, practitioners and athletes – Understand the barriers to and enablers of effective implementation of injury prevention programs in different populations

ACL, anterior cruciate ligament

## Supplementary Information

Below is the link to the electronic supplementary material.

Supplementary file1 (PDF 288 KB)
