# Supplementary material for: An Evidence-Based and Mechanistic Approach to Reducing the Risk of Anterior Cruciate Ligament Injury: An Exercise and Sport Science Australia Position Statement
Source: Sports Med. 2026 May 14;56(10):2389–411. doi: 10.1007/s40279-026-02450-3 (PMC13615120; doi:10.1007/s40279-026-02450-3)
Supplement: Supplementary file 1 [file 40279_2026_2450_MOESM1_ESM.pdf]

SUPPLEMENTARY MATERIAL

An evidence-based and mechanistic approach to reducing the risk of anterior cruciate ligament injury: an Exercise and Sport Science Australia position statement

Sports Medicine

Tyler J Collings[1,2], Laura E Diamond[1,2], Rod S Barrett[1,2], David J Saxby[1,2], David G Lloyd[1,2], Rod Whiteley[3], Brooke E Patterson[4,5], Kay M Crossley[4,5], Matthew N Bourne[1,2].

[1]Australian Centre for Precision Health and Technology (PRECISE), Griffith University, Gold Coast, QLD, Australia.

[2]School of Health Sciences and Social Work, Griffith University, Gold Coast, QLD, Australia.

[3]Rehabilitation Department, Aspetar Orthopaedic and Sports Medicine Hospital, Doha, Qatar

[4]La Trobe University Sport and Exercise Medicine Research Centre, La Trobe University, Melbourne, VIC, Australia.

[5]Australian IOC Research Centre, La Trobe University, Melbourne, VIC, Australia.

Corresponding author: Tyler Collings

Email: t.collings@griffith.edu.au

Address: 1 Parklands Dr, Southport QLD 4215, Gold Coast, Australia

1. Systematic literature search: Training intervention studies

Table 1. Search strategy keywords for intervention studies

PICOS framework	Search terms (joined with AND)
Population	player* OR athlete* OR sport* OR exercise OR "physical activity"
Intervention	"prevention program*" OR "prevention training" OR "training program*" OR "exercise program*" OR "training intervention" OR "neuromuscular training" OR "warm-up program*" OR "conditioning program*" OR "risk reduction" OR "plyometric training" OR "balance training" OR "strength training" OR "mobility training"
Comparison	-
Outcome	ACL OR "anterior cruciate ligament"
	injur* OR tear* OR rupture OR sprain*
Study design	"randomised controlled trial" OR "cluster-randomised" OR RCT OR prospective OR cohort OR longitudinal OR "training program*"

Inclusion criteria

- Exercise-based training intervention
- Control group consisting of no additional training or training as usual
- Either randomised or non-randomised by clusters or individuals
- Reported ACL injury rates for each group
- Peer-reviewed journal articles

Exclusion criteria

- No separate control group
- Less than 5 ACL injuries in total
- Conference posters or abstracts

Data extraction

- Program name
- Participant's sex, sport, age, and level
- Number of participants in the exercise and control groups
- Number of ACL injuries in the exercise and control groups
- Relative risk or injury rates/incidence per group
- Study design

Table 2. Articles meeting the inclusion and exclusion criteria.

Title	First Author	Year	Journal	DOI
Does the FIFA 11+ injury prevention program reduce the incidence of ACL injury in male soccer players?	Silvers-Granelli, H.J.	2017	Clinical Orthopaedics and Related Research	10.1007/s11999-017-5342-5
Prevention of acute knee injuries in adolescent female football players: cluster randomised controlled trial	Waldén, M.	2012	BMJ (Online)	10.1136/bmj.e3042
A randomized controlled trial to prevent noncontact anterior cruciate ligament injury in female collegiate soccer players	Gilchrist, J.	2008	American Journal of Sports Medicine	10.1177/0363546508318188
Lack of effect of a knee ligament injury prevention program on the incidence of noncontact anterior cruciate ligament injury	Pfeiffer, R.P.	2006	Journal of Bone and Joint Surgery	10.2106/JBJS.E.00616
A controlled prospective case control study of a prevention training program in female team handball players: the german experience.	Petersen, W.	2005	Archives of orthopaedic and trauma surgery	
Effectiveness of a neuromuscular and proprioceptive training program in preventing anterior cruciate ligament injuries in female athletes: 2-year follow-up	Mandelbaum, B.R.	2005	American Journal of Sports Medicine	10.1177/0363546504272261
Balance board training: prevention of traumatic injuries of the lower extremities in female soccer players? A prospective randomized intervention study.	Söderman, K.	2000	Knee surgery, sports traumatology, arthroscopy : official journal of the ESSKA	10.1007/s00402-005-0793-7
Avoidance of soccer injuries with preseason conditioning	Heidt Jr., R.S.	2000	American Journal of Sports Medicine	10.1177/03635465000280050601
Prevention of anterior cruciate ligament injuries in soccer. A prospective controlled study of proprioceptive training.	Caraffa, A.	1996	Knee surgery, sports traumatology, arthroscopy : official journal of the ESSKA	
Preventing injuries in female youth football–a cluster-randomized controlled trial	Steffen, Kathrin	2008	Scandinavian journal of medicine & science in sports	10.1111/j.1600-0838.2007.00703.x
Prevention of soccer-related knee injuries in teenaged girls	Kiani, Ashkan	2010	Archives of internal medicine	

Table 3. Methodology quality assessed using pooled PEDro scores extracted from previous systematic reviews.

	Source of PEDro score (previous systematic reviews)										
**Studies included in the review**	(Taylor et al., 2015)	(Sugimoto et al., 2015)	(Grindstaff et al., 2006)	(Myer et al., 2013)	(Gomes Neto et al., 2017)	(Al Attar et al., 2022)	(Van Beijsterveldt et al., 2013)	(Huang et al., 2020)	(Petushek et al., 2019)	Current authors	**Median rating**
Carraffa et al., (1996)										3	**3.0**
Mandelbaum et al., (2005)	3	3	5	3							**3.0**
Silvers-granelli et al., (2017)					6			7			**6.5**
Walden et al., (2012)	7	7		7		7		9	4		**7.0**
Kiani et al., (2010)	4	4		4							**4.0**
Petersen et al., (2005)	3	2	4	2					2		**2.0**
LaBella et al., (2011)	5	6		6		6		9			**6.0**
Gilchrist et al., (2008)	4	4		4		4		6			**4.0**
Heidt et al., (2000)	4	5		5			7		4		**5.0**
Steffen et al., (2008)	8	7		7	5	7	7	8	3		**7.0**
Pfeiffer et al., (2006)	3	2		2					2		**2.0**
Soderman et al., (2000)	4	4		4			4	5	0		**4.0**

2. Systematic literature search: Risk factor studies

Table 4. Search strategy keywords for risk factor studies

PICOS framework	Search terms (joined with AND)
Population	player OR athlete OR sport OR exercise OR "physical activity"
Intervention	-
Comparison	-
Outcome	acl OR “anterior cruciate ligament”
	"risk factor" OR "injury risk" OR "contributing factor" OR "predict*"
Study design	prospective OR longitudinal OR cohort OR case-control

Inclusion criteria

- Analysed and reported one or more intrinsic risk factor (e.g. odds ratio, relative risk, group comparison)
- Non-contact ACL injury mechanism
- Field and court sport population
- Peer reviewer journal article

Exclusion criteria

- Extrinsic risk factors only
- No univariable analysis of risk
- Snow-based sports, water-base sports, police, or military populations
- Less than 5 ACL injuries included in the analysis
- Conference posters or abstracts

Data extraction

- Statistically significant variables only

Table 5. Articles meeting inclusion and exclusion criteria

Title	First author	Year	Journal	DOI
Familial risk and its interaction with body mass index and physical activity in anterior cruciate ligament injury among first-degree relatives: a population-based cohort study	Ahn, H.S.	2021	The American journal of sports medicine	10.1177/03635465211032643
Posterior tibial slope as a risk factor for anterior cruciate ligament rupture in soccer players	Şenişik, S.	2011	Journal of Sports Science and Medicine	
Clinical-grade mri-based methods to identify combined anatomic factors that predict ACL injury risk in male and female athletes	Beaulieu, M.L.	2021	The American journal of sports medicine	10.1177/03635465211024249
Geometry of the articular cartilage of the tibial plateau is related to anterior cruciate ligament injury risk	Beynnon, B.	2011	Osteoarthritis and Cartilage	
Increased slope of the lateral tibial plateau subchondral bone is associated with greater risk of noncontact ACL injury in females but not in males: a prospective cohort study with a nested, matched case-control analysis.	Beynnon, B.D.	2014	The American journal of sports medicine	
Intrinsic risk factors for first-time noncontact ACL injury: a prospective study of college and high school athletes	Beynnon, B.D.	2023	Sports health	10.1177/19417381221121136
Do pediatric patients with anterior cruciate ligament tears have a higher rate of familial anterior cruciate ligament injury?	Bram, J.T.	2020	Orthopaedic Journal of Sports Medicine	10.1177/2325967120959665
Prior history of anterior cruciate ligament (ACL) reconstruction is associated with a greater risk of subsequent ACL injury in female collegiate athletes	Brumitt, J.	2019	Journal of Science and Medicine in Sport	10.1016/j.jsams.2019.08.005
Prediction of ACL-tear by lower limbs muscle strength and flexibility: a prospective cohort study in 95 female soccer players	Cejudo, A.	2024	Research in sports medicine (Print)	10.1080/15438627.2023.2280554
Strength and biomechanical risk factors for noncontact ACL injury in elite female footballers: a prospective study	Collings, T.J.	2022	Medicine and science in sports and exercise	10.1249/MSS.0000000000002908
Using an affordable motion capture system to evaluate the prognostic value of drop vertical jump parameters for noncontact ACL injury	Corban, J.	2023	The American journal of sports medicine	10.1177/03635465231151686
Increased lateral tibial slope is a risk factor for pediatric ACL injury: an mri-based case-control study of 152 patients	Dare, D.	2015	Orthopaedic Journal of Sports Medicine	10.1177/2325967115S00131
Does brain functional connectivity contribute to musculoskeletal injury? A preliminary prospective analysis of a neural biomarker of ACL injury risk	Diekfuss, J.A.	2019	Journal of Science and Medicine in Sport	10.1016/j.jsams.2018.07.004
Can two-dimensional video analysis during single-leg drop vertical jumps help identify non-contact knee injury risk? A one-year prospective study	Dingenen, B.	2015	Clinical Biomechanics	10.1016/j.clinbiomech.2015.06.013
Postural stability during single-leg stance: a preliminary evaluation of noncontact lower extremity injury risk	Dingenen, B.	2016	The Journal of orthopaedic and sports physical therapy	10.2519/jospt.2016.6278
Prospective correlation between serum relaxin concentration and anterior cruciate ligament tears among elite collegiate female athletes.	Dragoo, J.L.	2011	The American journal of sports medicine	
Analysis of biomechanical factors contributing to ACL injuries.	Dudhedia, U.	2024	Research Journal of Pharmaceutical, Biological and Chemical Sciences	10.33887/rjpbcs/2023.15.1.48
Baseline time to stabilization identifies anterior cruciate ligament rupture risk in collegiate athletes	DuPrey, K.M.	2016	The American journal of sports medicine	10.1177/0363546516629635
Poor validity of functional performance tests to predict knee injury in female soccer players with or without anterior cruciate ligament reconstruction	Fältström, A.	2021	The American journal of sports medicine	10.1177/03635465211002541
The familial predisposition toward tearing the anterior cruciate ligament: a case control study	Flynn, R.K.	2005	American Journal of Sports Medicine	10.1177/0363546504265678
Application of a clinic-based algorithm as a tool to identify female athletes at risk for anterior cruciate ligament injury: a prospective cohort study with a nested, matched case-control analysis.	Goetschius, J.	2012	The American journal of sports medicine	

Narrow notch width and low anterior cruciate ligament volume are risk factors for anterior cruciate ligament injury: a magnetic resonance imaging-based study	Gupta, R.	2022	HSS Journal	10.1177/15563316211041090
Risk factors for acute knee injury in female youth football	Hägglundd, M.	2016	Knee Surgery, Sports Traumatology, Arthroscopy	10.1007/s00167-015-3922-z
Shallow medial tibial plateau and steep medial and lateral tibial slopes: new risk factors for anterior cruciate ligament injuries	Hashemi, J.	2010	American Journal of Sports Medicine	10.1177/0363546509349055
Biomechanical measures of neuromuscular control and valgus loading of the knee predict anterior cruciate ligament injury risk in female athletes: a prospective study	Hewett, T.E.	2005	American Journal of Sports Medicine	10.1177/0363546504269591
Association between lower extremity muscular strength and acute knee injuries in young team-sport athletes	Hietamo, J.	2020	Translational Sports Medicine	10.1002/tsm2.172
Relationship between anterior cruciate ligament rupture and the posterior tibial and meniscal slopes in professional soccer athletes	Ikawa, M.H.	2021	Skeletal Radiology	10.1007/s00256-021-03776-x
Predicting ACL injury using machine learning on data from an extensive screening test battery of 880 female elite athletes	Jauhiainen, S.	2022	The American journal of sports medicine	10.1177/03635465221112095
Hip muscle strength predicts noncontact anterior cruciate ligament injury in male and female athletes: a prospective study	Khayambashi, K.	2016	The American journal of sports medicine	10.1177/0363546515616237
Type I collagen î±1 sp1 polymorphism and the risk of cruciate ligament ruptures or shoulder dislocations	Khoschnau, S.	2008	American Journal of Sports Medicine	10.1177/0363546508320805
Eln and fbn2 gene variants as risk factors for two sports-related musculoskeletal injuries	Khoury, L.E.	2015	International journal of sports medicine	10.1055/s-0034-1390492
Three genes associated with anterior and posterior cruciate ligament injury: a genome-wide association analysis	Kim, S.K.	2021	Bone and Joint Open	10.1302/2633-1462.26.BJO-2021-0040.R1
Psychological traits regarding competitiveness are related to the incidence of anterior cruciate ligament injury in high school female athletes	Kosaka, M.	2016	Knee	10.1016/j.knee.2016.04.015
The vertical drop jump is a poor screening test for ACL injuries in female elite soccer and handball players	Krosshaug, T.	2016	American Journal of Sports Medicine	10.1177/0363546515625048
Non-contact anterior cruciate ligament and lower extremity injury risk prediction using functional movement screen and knee abduction moment: an epidemiological observation of female intercollegiate athletes.	Landis SE	2018	Int J Sports Phys Ther	
Femoral intercondylar notch stenosis and correlation to anterior cruciate ligament injuries: a prospective study	Laprade, R.F.	1994	The American Journal of Sports Medicine	10.1177/036354659402200208
Is pre-injury leg length discrepancy a risk factor for anterior cruciate ligament injury in the skeletally immature athlete?	Lazaro, L.E.	2017	Orthopaedic Journal of Sports Medicine	10.1177/2325967117S00437
Altered hip control during a standing knee-lift test is associated with increased risk of knee injuries	Leppänenn, M.	2020	Scandinavian journal of medicine & science in sports	10.1111/sms.13626
Change of direction biomechanics in a 180-degree pivot turn and the risk for noncontact knee injuries in youth basketball and floorball players	Leppänen, M.	2021	The American journal of sports medicine	10.1177/03635465211026944
Sagittal plane hip, knee, and ankle biomechanics and the risk of anterior cruciate ligament injury: a prospective study	Leppänen, M.	2017	Orthopaedic Journal of Sports Medicine	10.1177/2325967117745487
Stiff landings are associated with increased ACL injury risk in young female basketball and floorball players	Leppänenn, M.	2017	The American journal of sports medicine	10.1177/0363546516665810
Intercondylar notch stenosis is not a risk factor for anterior cruciate ligament tears in professional male basketball players: an 11-year prospective study	Lombardo, S.	2005	American Journal of Sports Medicine	10.1177/0363546504266482
Are MMP3, MMP8 and TIMP2 gene variants associated with anterior cruciate ligament rupture susceptibility?	Lulińska-Kuklik, E.	2019	Journal of Science and Medicine in Sport	10.1016/j.jsams.2019.01.014
I spy with my little eye: a knee about to go 'pop'? Can coaches and sports medicine professionals predict who is at greater risk of ACL rupture?	Mørtvedt, A.I.	2020	British journal of sports medicine	10.1136/bjsports-2019-100602
Analysis of hip strength and mobility as injury risk factors in amateur women's soccer: a pilot study	Maestro, A.	2017	Archivos de Medicina del Deporte	

Intercondylar notch width and inner angle of lateral femoral condyle as the risk factors for anterior cruciate ligament injury in female handball players in herzegovina	Miljko, M.	2012	Collegium Antropologicum	
Development and validation of a clinic-based prediction tool to identify female athletes at high risk for anterior cruciate ligament injury.	Myer, G.D.	2010	The American journal of sports medicine	
Sex comparison of familial predisposition to anterior cruciate ligament injury	Myer, G.D.	2014	Knee Surgery, Sports Traumatology, Arthroscopy	10.1007/s00167-013-2822-3
The effects of generalized joint laxity on risk of anterior cruciate ligament injury in young female athletes	Myer, G.D.	2008	American Journal of Sports Medicine	
The relationship of hamstrings and quadriceps strength to anterior cruciate ligament injury in female athletes	Myer, G.D.	2009	Clinical Journal of Sport Medicine	10.1097/JSM.0b013e318190bddb
High body mass index and hip abductor muscle strength were risk factors for non-contact ACL injury in female high school basketball players: a prospective three-year cohort study	Nakase, J.	2017	Orthopaedic Journal of Sports Medicine	10.1177/2325967117S00319
Risk factors for noncontact anterior cruciate ligament injury in female high school basketball and handball players: a prospective 3-year cohort study	Nakase, J.	2020	Asia-Pacific Journal of Sports Medicine, Arthroscopy, Rehabilitation and Technology	10.1016/j.asmart.2020.06.002
Injury risk in collegiate football players with generalized joint hypermobility: a prospective cohort study over 2 years	Nicolay, R.W.	2023	Orthopaedic Journal of Sports Medicine	10.1177/23259671231167117
Increased posterior tibial slope and its association with ACL rupture in the pediatric population	O'Malley, M.P.	2014	Orthopaedic Journal of Sports Medicine	10.1177/2325967114S00093
Poor static balance is a novel risk factor for non-contact anterior cruciate ligament injury	Oshima, T.	2015	Orthopaedic Journal of Sports Medicine	10.1177/2325967115S00110
The landing error scoring system as a screening tool for an anterior cruciate ligament injury-prevention program in elite-youth soccer athletes	Padua, D.A.	2015	Journal of athletic training	10.4085/1062-6050-50.1.10
Two-dimensional and three-dimensional biomechanical factors during 90° change of direction are associated to non-contact ACL injury in female soccer players	Paolo, S.D.	2023	International Journal of Sports Physical Therapy	10.26603/001c.84308
Drop jump? Single-leg squat? Not if you aim to predict anterior cruciate ligament injury from real-time clinical assessment: a prospective cohort study involving 880 elite female athletes	Petushek, E.	2021	The Journal of orthopaedic and sports physical therapy	10.2519/jospt.2021.10170
Prediction of ACL injuries from vertical jump kinetics in division 1 collegiate athletes	Pontillo, M.	2021	International Journal of Sports Physical Therapy	10.26603/001c.18819
Genetic risk factors for anterior cruciate ligament ruptures: COL1A1 gene variant	Posthumus, M.	2009	British Journal of Sports Medicine	10.1136/bjsm.2008.056150
The COL5A1 gene is associated with increased risk of anterior cruciate ligament ruptures in female participants	Posthumus, M.	2009	American Journal of Sports Medicine	10.1177/0363546509338266
Investigation of angiogenesis genes with anterior cruciate ligament rupture risk in a south african population	Rahim, M.	2018	Journal of Sports Sciences	10.1080/02640414.2017.1322710
Increased lateral tibial plateau slope predisposes male college football players to anterior cruciate ligament injury	Rahnemai-Azar, A.A.	2016	Journal of Bone and Joint Surgery - American Volume	10.2106/JBJS.15.01163
Sex differences in the association between risk of anterior cruciate ligament rupture and col5a1 polymorphisms in elite footballers	Rodas, G.	2023	Genes	10.3390/genes14010033
Weaker lower extremity muscle strength predicts traumatic knee injury in youth female but not male athletes	Ryman Augustsson, S.	2017	BMJ Open Sport and Exercise Medicine	10.1136/bmjsem-2017-000222
Risk factors for leg injuries in female soccer players: a prospective investigation during one out-door season.	Södermann, K.	2001	Knee surgery, sports traumatology, arthroscopy : official journal of the ESSKA	
Greater body mass index and hip abduction muscle strength predict noncontact anterior cruciate ligament injury in female japanese high school basketball players	Shimozaki, K.	2018	Knee surgery, sports traumatology, arthroscopy : official journal of the ESSKA	10.1007/s00167-018-4888-4
No association between risk of anterior cruciate ligament rupture and selected candidate collagen gene variants in female elite athletes from high-risk team sports	Sivertsen, E.A.	2019	The American journal of sports medicine	10.1177/0363546518808467

Is knee neuromuscular activity related to anterior cruciate ligament injury risk? A pilot study	Smeets, A.	2019	Knee	10.1016/j.knee.2018.10.006
A prospective evaluation of the landing error scoring system (LESS) as a screening tool for anterior cruciate ligament injury risk.	Smith, H.C.	2012	The American journal of sports medicine	
Intercondylar notch size and anterior cruciate ligament injuries in athletes. A prospective study	Souryal, T.O.	1993	American Journal of Sports Medicine	10.1177/036354659302100410
Association between lower extremity muscle strength and noncontact ACL injuries	Steffen, K.	2016	Medicine and Science in Sports and Exercise	10.1249/MSS.0000000000001014
No association between static and dynamic postural control and ACL injury risk among female elite handball and football players: a prospective study of 838 players	Steffen, K.	2017	British journal of sports medicine	10.1136/bjsports-2016-097068
A decreased volume of the medial tibial spine is associated with an increased risk of suffering an anterior cruciate ligament injury for males but not females.	Sturnick, D.R.	2014	Journal of orthopaedic research : official publication of the Orthopaedic Research Society	10.1002/jor.22670
Lower hamstring to quadriceps muscle strength ratio and lower body weight as factors associated with noncontact anterior cruciate ligament injury in male american football players: a prospective cohort study	Taketomi, S.	2024	Asia-Pacific Journal of Sports Medicine, Arthroscopy, Rehabilitation and Technology	10.1016/j.asmart.2023.11.006
Meniscal bone angle is a strong predictor of anterior cruciate ligament injury	Teixeira Gonçalves Alves, L.F.	2022	Arthroscopy, Sports Medicine, and Rehabilitation	10.1016/j.asmr.2022.08.008
Bone morphology and morphometry of the lateral femoral condyle is a risk factor for ACL injury	Vasta, S.	2018	Knee surgery, sports traumatology, arthroscopy : official journal of the ESSKA	10.1007/s00167-017-4761-x
High risk of new knee injury in elite footballers with previous anterior cruciate ligament injury	Waldén, M.	2006	British Journal of Sports Medicine	10.1136/bjsm.2005.021055
Relationship between the risk of suffering a first-time noncontact ACL injury and geometry of the femoral notch and ACL: a prospective cohort study with a nested case-control analysis	Whitney, D.C.	2014	The American journal of sports medicine	10.1177/0363546514534182
Tibial spine volume is smaller in ACL-injured athletes compared to healthy athletes	Yahagi, Y.	2024	Knee Surgery, Sports Traumatology, Arthroscopy	10.1002/ksa.12161
Deficits in neuromuscular control of the trunk predict knee injury risk: a prospective biomechanical-epidemiologic study	Zazulak, B.T.	2007	American Journal of Sports Medicine	10.1177/0363546507301585
First-time anterior cruciate ligament injury in adolescent female elite athletes: a prospective cohort study to identify modifiable risk factors	Zebis, M.K.	2022	Knee surgery, sports traumatology, arthroscopy : official journal of the ESSKA	10.1007/s00167-021-06595-8
Identification of athletes at future risk of anterior cruciate ligament ruptures by neuromuscular screening	Zebis, M.K.	2009	American Journal of Sports Medicine	10.1177/0363546509335000

Al Attar, W. S. A., Bakhsh, J. M., Khaledi, E. H., Ghulam, H., & Sanders, R. H. (2022). Injury prevention programs that include plyometric exercises reduce the incidence of anterior cruciate ligament injury: a systematic review of cluster randomised trials. *Journal of physiotherapy*, *68*(4), 255-261.

Gomes Neto, M., Conceição, C. S., de Lima Brasileiro, A. J. A., de Sousa, C. S., Carvalho, V. O., & de Jesus, F. L. A. (2017). Effects of the FIFA 11 training program on injury prevention and performance in football players: a systematic review and meta-analysis. *Clinical Rehabilitation*, *31*(5), 651-659.

Grindstaff, T. L., Hammill, R. R., Tuzson, A. E., & Hertel, J. (2006). Neuromuscular control training programs and noncontact anterior cruciate ligament injury rates in female athletes: a numbers-needed-to-treat analysis. *Journal of Athletic Training (National Athletic Trainers' Association)*, *41*(4), 450-456. http://libraryproxy.griffith.edu.au/login?url=https://search.ebscohost.com/login.aspx?direct=true&db=ccm&AN=106249622&site=ehost-live&scope=site

https://www.ncbi.nlm.nih.gov/pmc/articles/PMC1748422/pdf/i1062-6050-41-4-450.pdf

Huang, Y.-L., Jung, J., Mulligan, C. M., Oh, J., & Norcross, M. F. (2020). A majority of anterior cruciate ligament injuries can be prevented by injury prevention programs: a systematic review of randomized controlled trials and cluster–randomized controlled trials with meta-analysis. *The American Journal of Sports Medicine*, *48*(6), 1505-1515.

Myer, G. D., Sugimoto, D., Thomas, S., & Hewett, T. E. (2013). The influence of age on the effectiveness of neuromuscular training to reduce anterior cruciate ligament injury in female athletes: a meta-analysis. *The American Journal of Sports Medicine*, *41*(1), 203-215.

Petushek, E. J., Sugimoto, D., Stoolmiller, M., Smith, G., & Myer, G. D. (2019). Evidence-based best-practice guidelines for preventing anterior cruciate ligament injuries in young female athletes: a systematic review and meta-analysis. *The American Journal of Sports Medicine*, *47*(7), 1744-1753.

Sugimoto, D., Myer, G. D., Foss, K. D. B., & Hewett, T. E. (2015). Specific exercise effects of preventive neuromuscular training intervention on anterior cruciate ligament injury risk reduction in young females: meta-analysis and subgroup analysis. *British Journal of Sports Medicine*, *49*(5), 282-289.

Taylor, J. B., Waxman, J. P., Richter, S. J., & Shultz, S. J. J. B. J. S. M. (2015). Evaluation of the effectiveness of anterior cruciate ligament injury prevention programme training components: a systematic review and meta-analysis. *49*(2), 79-87.

Van Beijsterveldt, A., Van Der Horst, N., Van De Port, I. G., & Backx, F. J. (2013). How effective are exercise-based injury prevention programmes for soccer players? A systematic review. *Sports Medicine*, *43*, 257-265.
